# Anaphylactic risk related to omalizumab, benralizumab, reslizumab, mepolizumab, and dupilumab

**DOI:** 10.1002/clt2.12038

**Published:** 2021-06-03

**Authors:** Lisha Li, Zixi Wang, Le Cui, Yingyang Xu, Kai Guan, Bin Zhao

**Affiliations:** ^1^ Department of Allergy Peking Union Medical College Hospital Chinese Academy of Medical Sciences & Peking Union Medical College Beijing Key Laboratory of Precision Medicine for Diagnosis and Treatment on Allergic Diseases National Clinical Research Center for Dermatologic and Immunologic Diseases Beijing China; ^2^ Department of Pharmacy Peking Union Medical College Hospital Chinese Academy of Medical Sciences & Peking Union Medical College Beijing China

**Keywords:** anaphylaxis, life‐threatening outcomes, monoclonal antibodies, pharmacovigilance study, severe asthma

## Abstract

**Background:**

Monoclonal antibodies (mAbs) are novel, effective therapeutics for the treatment of inadequately controlled severe asthma. Knowledge of the anaphylaxis risks related to different mAbs is essential for their appropriate and safe administration. This study aimed to evaluate the associations between different mAbs and anaphylactic reactions by applying statistical approaches to pharmacovigilance data.

**Methods:**

This was a retrospective study using data from the US Food and Drug Administration Adverse Event Reporting System database from January 2004 to September 2020. A total of 2006 reports of anaphylaxis related to benralizumab, dupilumab, mepolizumab, omalizumab, and reslizumab were obtained through data mining. The clinical characteristics of the cases were analyzed, and the risk signals of anaphylactic reactions and corresponding outcomes were investigated in the five mAbs.

**Results:**

The patients were mainly young and middle‐aged adults, with markedly more women than men. Omalizumab, benralizumab, reslizumab, and mepolizumab showed positive signals for anaphylaxis, while only dupilumab showed a negative signal. The risk of initial or prolonged hospitalization due to anaphylaxis was significantly higher in the benralizumab group than in the omalizumab group (42.86% vs. 28.92%, *p* = 0.024). Further, when anaphylaxis to omalizumab occurred, patients with asthma were more likely to have life‐threatening outcomes than those with chronic urticaria (18.0% vs. 12.9%, *p* = 0.022).

**Conclusion:**

In the current real‐world study, the positive anaphylaxis signals related to omalizumab, benralizumab, reslizumab, and mepolizumab suggested the need for the close monitoring of patients after drug use, and dupilumab showed a negative signal for anaphylaxis.

## 1 BACKGROUND

Asthma is a major public health problem worldwide, with high morbidity, mortality, and heavy economic burden.^1^ Despite extensive efforts, there is still a small proportion of patients with severe asthma insufficiently controlled with high‐dose inhaled corticosteroids and are oral corticosteroid‐dependent.^2^ Specific monoclonal antibodies (mAbs) are novel therapeutic agents against severe asthma and can significantly reduce disease burden and asthma mortality.^3^ Several mAbs, including benralizumab, dupilumab, mepolizumab, omalizumab, and reslizumab, have been approved as add‐on maintenance therapeutics for severe, inadequately controlled eosinophilic asthma.

As the number of mAbs used for asthma increases increased knowledge of risks of hypersensitivity and life‐threatening anaphylaxis associated with different mAbs is critical for their appropriate and safe administration. Premarketing clinical trial data of omalizumab showed an anaphylaxis incidence of less than 0.1% in 3854 subjects; however, post‐marketing surveillance data from the Food and Drug Administration (FDA) showed that the frequency of anaphylaxis was more than 0.2% in patients receiving omalizumab.^4^ Several clinical trials have shown a high level of safety of mepolizumab and no case of anaphylaxis has been reported,^5^ while 0.3% of patients developed anaphylaxis in the preapproval clinical program of reslizumab.^6^

At present, safety data for the five mAbs remain insufficient, necessitating long‐term monitoring. Further, studies evaluating the risk of anaphylaxis with these mAbs are still lacking. This study aimed to investigate the correlations between different mAbs and anaphylactic reactions by applying statistical approaches to a pharmacovigilance database and comparing the rate of adverse outcomes following anaphylaxis.

## 2 METHODS

### 2.1 Data source

This was a retrospective pharmacovigilance study using the US Food and Drug Administration Adverse Event Reporting System (FAERS) database from January 2004 to September 2020. The FAERS database is a spontaneous reporting system that contains information about drug adverse reactions and error reports of medication submitted by physicians, nurses, patients, and manufacturers. The FAERS database comprises seven datasets, including patient demographic and administrative information (DEMO), drug information (DRUG), report sources (RPSR), indications for drug administration (INDI), adverse events (REAC), therapy start and end dates for reported drugs (THER), and patient outcomes (OUTC).

### 2.2 Data mining for signal detection of anaphylactic adverse drug reactions

The Preferred Term “anaphylactic reaction” (code: 10002198) was used to identify anaphylactic events. From the REAC files according to the Medical Dictionary for Regulatory Activities (MedDRA, version 23.1). In the data mining process, IBM Micromedex (IBM Corp., Armonk) was used as a dictionary to select the generic and brand names of mAbs, including omalizumab (Xolair or Xolair PFS), benralizumab (Fasenra or Fasenra Pen), dupilumab (Dupixent), mepolizumab (Nucala), and reslizumab (Cinqaero or Cinqair).

**FIGURE 1:**
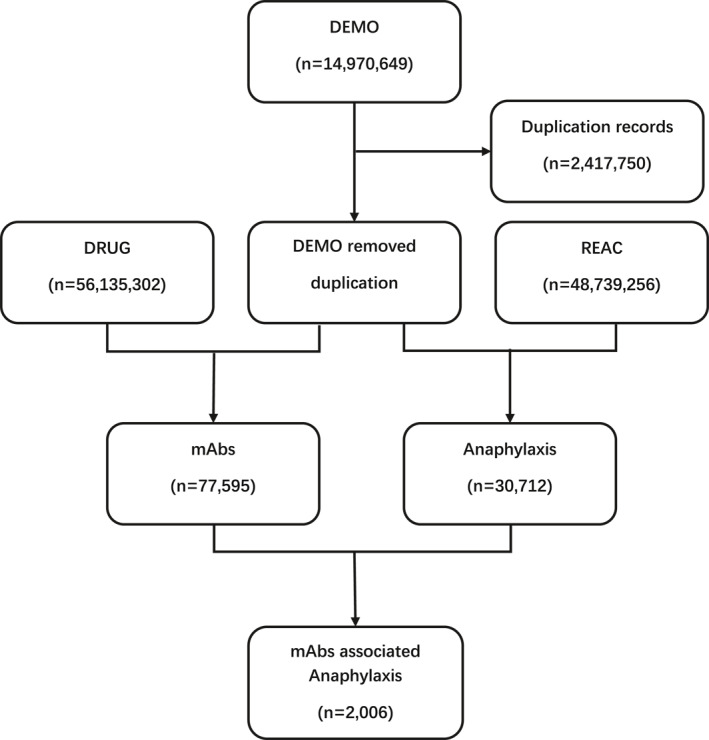
Data mining algorithm for signal detection of anaphylaxis cases related to the five monoclonal antibodies. DEMO, dataset of patient demographic and administrative information; DRUG, dataset of drug information; mAbs, monoclonal antibodies; REAC, dataset of drug adverse events

We obtained 14,970,649 reports from the FAERS database and reduced the number of reports to 12,552,899 after removing the duplicated records based on the FDA recommendations. Finally, we identified 2006 reports of anaphylaxis associated with the five mAbs (Figure 1).

### 2.3 Statistical analysis

All data mining and statistical analyses were performed using SAS software (ver. 9.4; SAS Institute Inc., Cary, NC). To evaluate the association between different mAbs and anaphylactic events, four algorithms based on the Bayesian analysis and non‐proportional analysis were adopted, namely, the reporting odds ratio (ROR), proportional reporting ratio (PRR), Bayesian confidence propagation neural network, and multi‐item gamma Poisson shrinker algorithm.^7^, ^8^, ^9^, ^10^, ^11^, ^12^, ^13^, ^14^ The normality of the data was examined using the Kolmogorov‐Smirnov test. Pearson’s *χ*2 test was used to compare the rate of adverse outcomes following anaphylaxis between different mAbs. The correlations between some factors and life‐threatening outcomes associated with omalizumab‐induced anaphylaxis were estimated using Pearson *χ*2 or Mann‐Whitney U tests. Differences were considered statistically significant at *p*‐values less than 0.05.

## 3 RESULTS

### 3.1 Clinical characteristics of anaphylaxis cases related to mAbs

A total of 2006 cases of anaphylaxis associated with the five mAbs were identified. The demographic information of patients reporting anaphylaxis related to the five mAbs is listed in Table 1. Most patients were young and middle‐aged adults, with children and people older than 65 years constituting a small proportion of the entire cohort. The number of female patients was 5–10 times that of male patients in the anaphylaxis cases related to omalizumab, benralizumab, and mepolizumab, while all the four anaphylaxis cases related to reslizumab were female.

**TABLE 1:** Demographic characteristics of the cases reporting anaphylaxis related to the five monoclonal antibodies

Indexes	Omalizumab	Benralizumab	Dupilumab	Mepolizumab	Reslizumab	Total
Age(years old)						
<18	135	1	5	2		143
18–44	438	12	20	22	2	494
45–64	284	20	8	24	2	338
≥65	50	10	1	6		67
Unknown age	866	20	28	50		964
Gender						
Female	1246	52	27	74	4	1403
Male	261	5	12	15		293
Unknown gender	266	6	23	15		310
Report year						
2004–2009	243					243
2010–2014	452					452
2015–2017	546		1	20	2	569
2018–2020	532	63	61	84	2	742

The distribution of adverse event report dates was affected by the duration of drug availability in the market. For instance, omalizumab has been in the market for nearly 20 years and was associated with the largest number of anaphylaxis cases, with reports increasing every year. Indications for drug use are shown in Table 2. All five mAbs were mainly used in patients with asthma; however, omalizumab was additionally used in chronic urticaria and dupilumab in atopic dermatitis.

**TABLE 2:** Indications for the use of the five monoclonal antibodies

Indications	Omalizumab	Benralizumab	Dupilumab	Mepolizumab	Reslizumab	Total
Asthma	857	47	19	69	2	994
Chronic urticaria	409					409
Anaphylactic reaction	23					23
Food allergy	8					8
Mastocytosis	7					7
Inflammation	6					6
Atopic dermatitis	5		23			28
Allergy to arthropod sting	4					4
Hypersensitivity	4					4
Mast cell activation syndrome	4					4
Allergic rhinitis	4					4
Rubber sensitivity	3					3
Bronchitis	1					1
Multiple allergies	1					1
Skin test	1					1
Aspirin‐exacerbated respiratory disease			2			2
Chronic eosinophilic Pneumonia				1		1
Nasal polyps			1			1
Unknown indication	427		1	32		460

### 3.2 Anaphylaxis related to different mAbs

The anaphylaxis signals for the five mAbs were analyzed using four algorithms, as shown in Table 3. Omalizumab showed the highest ROR, PRR, information component (IC), and empirical Bayes geometric mean (EBGM). Further, benralizumab, mepolizumab, and reslizumab also showed positive signals of anaphylactic reactions, but their signals including ROR, PRR, IC, and EBGM were relatively lower than those of omalizumab. Only dupilumab showed completely negative signals for anaphylaxis among the five mAbs.

**TABLE 3:** Comparison of anaphylaxis signals related to different monoclonal antibodies

	*N*	ROR	PRR	IC	EBGM
		(95% Two‐sided CI)	(*χ*2)	(IC025)	(EBGM05)
Omalizumab	1773	24.19(23.03,25.41)	22.96(35175.62)	4.44(4.23)	21.69(20.82)
Benralizumab	63	8.48(6.6,10.88)	8.32(406.11)	3.05(2.38)	8.31(6.74)
Dupilumab	62	0.8(0.63,1.03)	0.8(2.99)	−0.32(−)	0.8(0.65)
Mepolizumab	104	4.65(3.83,5.64)	4.61(293.3)	2.2(1.81)	4.59(3.91)
Reslizumab	4	5.74(2.14,15.41)	5.68(15.46)	2.51(0.93)	5.68(2.49)

*Note:* Criteria of positive signals: ROR, 95% CI > 1, *N* ≥ 2; PRR, PRR ≥ 2, *χ*2 ≥ 4, *N* ≥ 3; IC, IC025 > 0; EBGM, EBGM05 > 2, *N* > 0.

Abbreviations: CI, confidence interval; EBGM, empirical Bayesian geometric mean; EBGM05, the lower 90% one‐sided CI of EBGM; IC, information component; IC025, the lower limit of the 95% two‐sided CI of the IC; N, number; PRR, proportional reporting ratio; ROR, reporting odds ratio; χ2, chi‐squared.

### 3.3 Clinical outcomes of anaphylaxis related to mAbs

As listed in Table 4, the risk of death following anaphylaxis was low with all five mAbs; however, anaphylaxis to omalizumab was associated with disability in 14 cases. There was no significant difference in the proportion of life‐threatening events among the five mAbs; however, the risk of initial or prolonged hospitalization due to anaphylaxis appeared to be significantly higher in the benralizumab group than in the omalizumab group (42.86% vs. 28.92%, *p* = 0.024).

**TABLE 4:** Clinical outcomes of anaphylaxis related to different monoclonal antibodies

Outcome	Reports (*N*,%)				
	Omalizumab	Benralizumab	Dupilumab	Mepolizumab	Reslizumab
Death	5(0.28)	0(0.00)	1(1.61)	2(1.92)	0(0.00)
Disability	14(0.79)	0(0.00)	0(0.00)	0(0.00)	0(0.00)
Hospitalization ‐ initial or prolonged	511(28.92)	27(42.86)^a^	25(40.32)	31(29.81)	1(25)
Life‐threatening	255(14.43)	11(17.46)	9(14.52)	21(20.19)	1(25)
Other serious important medical event	1505(85.17)	74(117.46)	70(112.9)	127(122.12)	4(100)
Required intervention to prevent permanent impairment/damage	15(0.85)	0(0.00)	0(0.00)	0(0.00)	0(0.00)

^a^ The risk of initial or prolonged hospitalization due to anaphylaxis was significantly higher in the benralizumab group than in the omalizumab group, *p* = 0.024.

### 3.4 Factors associated with life‐threatening anaphylactic events related to omalizumab

In this study, among the 2006 cases of anaphylaxis, 1773 (88.4%) were related to omalizumab. These cases were further divided into subgroups with or without life‐threatening outcomes, and demographic characteristics and indications for drug use of the two subgroups were compared. The results suggested that age, sex ratio, and weight of patients were similar between the two subgroups (*p* > 0.5); however, in terms of indications for drug use, patients with asthma were more likely to have life‐threatening outcomes (18.0%) than patients with chronic urticaria (12.9%) when anaphylaxis to omalizumab occurred (*p* = 0.022).

## 4 DISCUSSION

This pharmacovigilance study mined data from the FAERS database and analyzed the characteristics of anaphylaxis cases related to benralizumab, dupilumab, mepolizumab, omalizumab, and reslizumab. Simultaneously, the reporting odds ratios of anaphylaxis and the corresponding clinical outcomes among the five mAbs were compared. As this was a real‐world study that analyzed postmarketing surveillance data, its results would be more representative of real experience in clinical practice than those of clinical trials. The large sample size—more than 2,000 anaphylaxis cases associated with the five mAbs—was another advantage.

The Bayesian analysis and non‐proportional analysis found that omalizumab, benralizumab, reslizumab, and mepolizumab showed positive signals for anaphylaxis, while only dupilumab showed a negative signal. This result is consistent with the literature reports. The risk of anaphylaxis with omalizumab was estimated to be 0.1%–0.2%,^15^ and the incidence of anaphylaxis caused by reslizumab in clinical trials was ∼0.3%;^16^ this prompted the FDA to issue a black box warning for both these drugs. Further, hypersensitivity reactions, including anaphylaxis, have been observed in patients receiving benralizumab.^17^ The relatively higher ROR detected with omalizumab, benralizumab, and reslizumab indicated the need for keeping patients informed and having a post‐injection observation period.^17^ As to mepolizumab, clinical trials of mepolizumab showed no cases of drug‐related anaphylaxis;^18^ however, our results found that mepolizumab had a low but positive anaphylaxis signal. In addition, a case of anaphylaxis following the administration of mepolizumab was recently reported by Jingo et al.,^19^ and more cases might be observed with the extension of the monitoring period. Thus, we also need to be aware of the risk of anaphylaxis with mepolizumab during its clinical application. In the current study, dupilumab was the only mAb with a negative anaphylaxis signal, which might be related to its degree of humanization. Among the five mAbs, dupilumab is the only fully human mAb with 99% human component, while the other four mAbs are humanized mAbs with 90% human components. Anaphylaxis reactions to humanized mAbs are not so common, but still exist because of the persistent immunogenicity caused by using transgenic mouse cell lines, which cannot generate human carbohydrate side chains;^20^ however, fully human mAbs do not have this defect. The good safety profile of dupilumab supports its self‐administration at home.

Further, the current study found that female patients made up a large part of anaphylaxis cases associated with the five mAbs; reports in the literature had a similar gender distribution. In a study reviewing spontaneous post‐marketing adverse event reports of omalizumab, most cases of anaphylaxis were female patients.^21^ Another recent study including 96 patients diagnosed with anaphylaxis caused by omalizumab revealed a preponderance of female subjects (84%).^22^ A pooled safety analysis from six trials of reslizumab identified three cases of anaphylaxis, all of which were women.^16^ As there is no sex preference for drug use, the female predominance suggested that female sex might be a potential risk factor for anaphylaxis related to these mAbs.

In the subsequent analysis, the indication of drug use showed some association with life‐threatening outcomes following an anaphylactic reaction to omalizumab, wherein patients with asthma were found to be more likely to have life‐threatening outcomes than patients with chronic urticaria. Asthma is a well‐recognized risk factor for severe anaphylaxis.^23^ After controlling for age, sex, race/ethnicity, comorbidities, and allergen immunotherapy, there was a 5.2‐fold increased incidence of anaphylactic shock in patients with asthma.^24^ In addition, most cases of fatal food anaphylaxis occurred in individuals with a history of asthma with a previous diagnosis of food allergy.^25^ Our results were consistent with these literature reports.

The major limitation of this study is that the analysis is based on data mined from a voluntary SRS, and thus data are limited by under‐reporting of cases, indeterminate causality, and the potential existence of duplicate reports;^26^ further, the definition and grading of anaphylaxis events recorded in the database are not well standardized or not enough accurate; therefore the data could only reflect some important trends in reports of anaphylaxis related to different mAbs, but could not be used to compare the incidence of anaphylaxis events among mAbs. Moreover, this data is mainly based on the US population, but prescription patterns or approval times of mAbs vary in different countries; therefore, the analysis conclusions could not be directly extrapolated to other countries. Another source of bias in this study is that mAbs with longer post‐marketing monitoring periods tend to have more reports of adverse events than those mAbs introduced recently. Thus the results of this study just provide some clues for the anaphylactic risk of the five mAbs, and further researches conducted in multiple countries later when these mAbs are widely used around the world for a long time would be necessary to verify the findings of the current study.

## 5 CONCLUSION

In the current real‐world study, omalizumab, benralizumab, reslizumab, and mepolizumab had positive signals for anaphylaxis, suggesting the need for close monitoring of patients after drug use. Increased attention and alerts are especially required for mepolizumab, which had almost no report of anaphylaxis in the literature. Only dupilumab showed a negative signal for anaphylaxis among the five mAbs.

## CONFLICT OF INTERESTS

The authors declare that there is no conflict of interests that could be perceived as prejudicing the impartiality of the research reported.

## AUTHOR CONTRIBUTIONS

Lisha Li: Data curation (lead), Conceptualization (supporting), Formal analysis (equal), Investigation (supporting).
